# Commentary: Anti-tumor Effect of Oleic Acid in Hepatocellular Carcinoma Cell Lines *via* Autophagy Reduction

**DOI:** 10.3389/fcell.2021.677595

**Published:** 2021-04-13

**Authors:** Jiong Lin

**Affiliations:** Department of Oncology, Affiliated Hospital of Guangdong Medical University, Zhanjiang, China

**Keywords:** oleic acid, hepatocellular carcinoma, invasion, cell death, migration

Recently, we read a paper in Frontiers in Cell and Developmental Biology “Anti-tumor Effect of Oleic Acid in Hepatocellular Carcinoma Cell Lines via Autophagy Reduction.” Oleic Acid (OA) may be a promising drug candidate for the treatment of hepatocellular carcinoma (HCC). However, some problems existing in this paper need to be commented.

With great interest we read a paper “Anti-tumor Effect of Oleic Acid in Hepatocellular Carcinoma Cell Lines via Autophagy Reduction” published in Frontiers in Cell and Developmental Biology (Giulitti et al., 2021). Blocking autophagy suppresses the growth of HCC cells (Zai et al., 2020). Authors indicated that OA induced cell death and reduced migration and invasion of the HCC cells, suggesting OA could be a promising drug candidate for the treatment of disease. However, some issues existing in this paper need to be commented.

In the paper, OA inhibited the invasion and migration of HCC cells after treatment with OA (300 μM) for 48 h (Figure 6). However, authors demonstrated that OA significantly suppressed cell proliferation and induced cell death of HCC cells after incubation with the same concentrations (300 μM) of OA for the same time (48 h) (Figures 3–5). These results strongly suggested that the cause responsible for inhibition of migration and invasion of cells could be due to apoptosis induction of OA. Authors could treat the cells with OA at a lower concentration or for a short time when studying the inherent effects of drugs on the migration and invasion ability of cells without apoptosis induction. This made the results more convincing.

## Author Contributions

JL: study concept and design, data analysis, methodology, drafting manuscript, review and editing, and supervision.

## Conflict of Interest

The author declares that the research was conducted in the absence of any commercial or financial relationships that could be construed as a potential conflict of interest.
